# Factors associated with post-stroke fatigue among stroke survivors: a cross-sectional study

**DOI:** 10.7717/peerj.19052

**Published:** 2025-03-06

**Authors:** Yuan Dong, Linxi Tang, Salwismawati Badrin, Salziyan Badrin, Jiarun Wu

**Affiliations:** 1 School of Health Sciences, Universiti Sains Malaysia, Kubang Kerian, Kelantan, Malaysia; 2 School of Medical Sciences, Universiti Sains Malaysia, Kubang Kerian, Kelantan, Malaysia; 3 School of Physical Health, Guizhou University of Traditional Chinese Medicine, Guiyang, Guizhou, China

**Keywords:** Post-stroke fatigue, Depression, Quality of life, Activities of daily living

## Abstract

**Background:**

Post-stroke fatigue (PSF) is a common complication experienced by stroke survivors. These individuals often confront psychological challenges such as depression and anxiety, along with significant obstacles like reduced quality of life (QoL) and limitations in activities of daily living (ADLs). Such challenges can profoundly affect their overall recovery and well-being. Despite its prevalence, the associated factors contributing to PSF remain poorly understood. This study aims to primarily investigate these associated factors, while also examining the interrelationships among PSF, depression level, QoL, and ADLs, highlighting the need for a better understanding of these complex interactions.

**Methods:**

This cross-sectional study involved 271 stroke survivors and was conducted at the Department of Neurology, Second Affiliated Hospital of Guizhou University of Traditional Chinese Medicine, China, from September 2023 to January 2024. Participants independently completed the Fatigue Severity Scale (FSS), Patient Health Questionnaire-9 (PHQ-9), and the Short Version of the Stroke-Specific Quality of Life Scale (SV-SS-QoL) as part of a convenience sampling method, while medical professionals assessed the Barthel Index (BI) using the same sampling framework. Multivariable linear regression analyses were employed to determine the factors associated with the persistence of PSF.

**Results:**

The mean FSS score was 35.04 ± 11.60, while the average score for the SV-SS-QoL was 34.28 ± 9.51, and the BI score averaged 77.79 ± 25.90. Approximately 45.8% of participants (*n* = 124) experienced PSF. The mean score on the PHQ-9 was 7.63 ± 6.13. A significant negative correlation was identified between fatigue and both QoL and ADLs (*P* < 0.01). Furthermore, multiple linear regression analyses revealed that age, gender, income level, depression level, QoL, and ADLs were associated factors of fatigue severity among stroke survivors, all showing statistically significant differences (*P* < 0.01).

**Conclusion:**

This study emphasizes the critical factors associated with PSF and highlights the necessity of developing targeted interventions, such as depression management, QoL enhancement, and ADLs restoration, to alleviate fatigue in stroke survivors. Future research should focus on evaluating the effectiveness of these interventions to optimize rehabilitation outcomes and improve survivors’ overall recovery and well-being.

## Introduction

In the context of rapid socio-economic development, significant lifestyle changes have emerged, including elevated stress levels, declining sleep quality, and the adoption of unhealthy dietary habits. These changes have contributed to a sharp rise in chronic conditions such as hypertension, diabetes, and hypercholesterolemia, significantly increasing the global burden of stroke (Hu, 2023; Tu & Wang, 2023; Yin et al., 2022). In 2020 alone, approximately 17.8 million new stroke cases were reported worldwide, underscoring the persistence of stroke as a major global health challenge (Yahya et al., 2020; Chau et al., 2023). While stroke has traditionally been associated with older adults, recent trends indicate a growing prevalence among younger populations, with individuals aged 40 to 64 now accounting for 66.6% of new stroke cases (Falkenstein, Karthaus & Brüne-Cohrs, 2020; Tu & Wang, 2023).

Post-stroke fatigue (PSF) is one of the most prevalent and persistent complications among stroke survivors, significantly hindering recovery. PSF is characterized by enduring physical and mental fatigue that does not resolve with adequate rest (Liu-Ambrose et al., 2022). It affects approximately 33 million stroke survivors globally, with reported prevalence rates ranging from 16% to 75%, largely due to variations in assessment tools and methodologies (Alghamdi et al., 2021; Chen et al., 2023; Kjeverud, 2024). Studies have shown that 56% of survivors experience fatigue symptoms within the first month post-stroke, with 45% continuing to report symptoms for over a decade (Damsbo et al., 2020; Vetkas et al., 2020; Western et al., 2020).

With the increasing prevalence of PSF and its considerable impact on stroke survivors, understanding its associated factors is essential. Age is recognized as a critical associated factor, as older adults are more vulnerable due to declining physiological resilience, age-related comorbidities, and slower recovery processes (Huang et al., 2024). Additionally, PSF is closely linked to depression, which creates a self-reinforcing negative cycle: persistent fatigue exacerbates emotional distress, while depressive symptoms further intensify PSF severity and delay recovery (Northcott et al., 2016). This cycle significantly disrupts activities of daily living (ADLs) and diminishes quality of life (QoL). Stroke survivors with PSF often face considerable difficulties in performing essential tasks such as dressing, preparing meals, and independent mobility, leading to reduced functional autonomy. Consequently, declines in QoL, reflected in diminished social participation and impaired emotional well-being, highlight the multidimensional burden of PSF and its far-reaching consequences for overall well-being (Ogwumike et al., 2022; Vollertsen et al., 2023).

Current research on PSF has mainly focused on elderly stroke survivors, whereas studies on young and middle-aged survivors who are able to ambulate independently remain limited. This group experiences combined pressures from work and family responsibilities, which can aggravate fatigue and mental stress, thereby worsening their ability to manage daily tasks and overall quality of life (Falkenstein, Karthaus & Brüne-Cohrs, 2020; Lei & Xia, 2023). Therefore, identifying the factors associated with PSF in this population is essential for designing effective interventions.

Our primary objective is to identify factors associated with PSF in stroke survivors, while our secondary aim is to explore the relationships between PSF, levels of depression, QoL, and ADLs, with the goal of deepening the understanding of the interactions among these variables. This study aims to identify factors associated with PSF in stroke survivors, with a focus on young and middle-aged stroke survivors, to provide a better understanding of PSF and inform future clinical management. We hypothesize that demographic factors such as age, gender, and income level are anticipated to be significantly associated with PSF prevalence. Furthermore, higher levels of depression are hypothesized to occur alongside greater PSF severity and are accompanied by lower ADLs and QoL.

## Methods

### Population and sample

This study employed a cross-sectional design to investigate stroke survivors. A convenience sample was drawn from 271 consecutive patients diagnosed with cerebral ischemia who were admitted to the Department of Neurology at the Second Affiliated Hospital of Guizhou University of Traditional Chinese Medicine in China between September 2023 and January 2024. Inclusion criteria for participation were: (1) aged between 18 and 60 years; (2) able to ambulate independently (not bed-ridden); and (3) diagnosed with stroke within the last three months. Individuals who were elderly, unconscious, or suffering from other neurological disorders were excluded from the study. Prior to the study’s initiation, a comprehensive literature review was conducted to determine the appropriate sample size. The calculation employed the single proportion formula outlined by Pourhoseingholi, Vahedi & Rahimzadeh (2013), aiming for a 95% confidence interval (CI) with a 5% margin of error. Based on a reported 20% prevalence of post-stroke fatigue by Vitturi et al. (2021), we determined that 246 participants would be required to meet the desired confidence level. To accommodate an anticipated non-response rate of 10%, the final sample size was adjusted to include 271 participants.

### Ethical consideration

The Medical Ethics Committee of the Second Affiliated Hospital of Guizhou University of Traditional Chinese Medicine, along with the Ethics Committee of Universiti Sains Malaysia (USM/JEPeM/KK/23090681), provided ethical approval for the study, adhering to the principles outlined in the Declaration of Helsinki.

### Assessment instruments

Our study employed a structured questionnaire comprising five sections. The first section, designed by the researcher, focused on sociodemographic characteristics derived from previous studies (Chen et al., 2023; Zeng et al., 2024), including variables such as age, gender, income level, education level, marital status, occupational status, and living situations.

The second section of the questionnaire utilized the Chinese version of the Fatigue Severity Scale (FSS) to evaluate post-stroke fatigue based on patients’ subjective experiences (Wu & Wang, 2007). This scale comprises nine items, with total scores ranging from 9 to 63 points. A score of 36 points or higher indicates the presence of post-stroke fatigue, with higher scores reflecting greater severity of fatigue.

The third section incorporated the Patient Health Questionnaire-9 (PHQ-9) in Chinese, which assesses depressive symptoms experienced by stroke survivors over the past two weeks (Wang et al., 2014). This scale also contains nine items and yields a maximum score of 27 points, with higher scores indicating increased levels of depression. Based on the total scores, the severity of depression was classified into five categories: no depression (0–4 points), mild (5–9 points), moderate (10–14 points), severe (15–19 points), and extremely severe (≥20 points).

In the fourth section, the Chinese version of the Short Version of the Stroke-Specific Quality of Life Scale (SV-SS-QoL) was administered to evaluate the QoL among stroke survivors. This scale includes two components that assess both physical and mental health (Tang et al., 2021). Comprising nine items, it has a total score range from 0 to 27 points, with higher scores indicating better QoL. The levels of QoL were categorized as low (12–27.99 points), moderate (28.00–43.99 points), and high (44.00–60.00 points).

Lastly, the Chinese version of the Barthel Index (BI) was used to assess patients’ ADLs (Hou et al., 2012). To maintain objectivity, healthcare professionals observed patients directly during evaluations. The BI consists of ten items, with a maximum score of 100 points, reflecting the individual’s self-care abilities. Higher scores denote greater independence, with classifications as follows: complete independence (100 points), mild dependence (61–99 points), moderate dependence (41–60 points), severe dependence (21–40 points), and extreme dependence (≤20 points).

### Data collection

During data collection, researchers initially screened participants to ensure they met the inclusion and exclusion criteria, confirming their eligibility. Once eligibility was established, the research team provided each participant with a thorough explanation of the study’s purpose and procedures, ensuring that they fully comprehended and were willing to participate. The written informed consent was then obtained in accordance with ethical standards. A comprehensive questionnaire was subsequently developed, covering multiple domains of information. Participants independently completed sections on sociodemographic characteristics and self-assessment scales, including the FSS, PHQ-9, and the SV-SS-QoL. In contrast, the BI was objectively assessed by healthcare professionals through direct observation of participant behavior. All questionnaires were administered in an environment that provided appropriate guidance and support as needed. This structured approach to data collection established a solid foundation for subsequent analysis, enhancing the validity and reliability of the findings.

### Statistical analysis

Data analysis was conducted using SPSS version 29.0. Descriptive statistics were used to summarize sociodemographic data and assess the prevalence of PSF among stroke survivors. The normality of the dataset was examined using P-P plots, skewness, and kurtosis, which confirmed that the data did not significantly deviate from a normal distribution. For group comparisons, independent samples t-tests were applied for pairwise comparisons, and one-way analysis of variance (ANOVA) was used for comparisons across multiple groups. To explore the relationships between the severity of fatigue, depression level, QoL, and ADLs, Pearson’s correlation analysis was performed. In addition, multiple linear regression analysis was conducted to identify factors associated with the severity of fatigue in stroke survivors. Multicollinearity among predictors was assessed using the variance inflation factor (VIF), with VIF < 5, indicating no significant collinearity issues. Statistical significance was set at *P* < 0.05.

## Results

### Internal consistency of measures

In this study, the internal consistency of the Fatigue Severity Scale (FSS), Patient Health Questionnaire-9 (PHQ-9), Stroke-Specific Quality of Life Scale (SV-SS-QoL), and Barthel Index (BI) was evaluated. The Cronbach’s *α* values were 0.946, 0.917, 0.906, and 0.936, respectively, indicating excellent reliability of the measures used.

### Sociodemographic characteristics

A total of 271 stroke survivors participated in the study, achieving a response rate of 100%. Participants with higher levels of fatigue severity were predominantly aged 40–59 years (86.3%), and females accounted for 47.2% of the sample. Additionally, 74.5% of participants reported an income level of ≤ CNY 3000, while the majority had an educational level limited to primary school (31.4%). Notable proportions of participants experiencing significant fatigue were widowed (7.4%), unemployed (13.7%), or living alone (7.7%) (Table 1).

**Table 1:** Sociodemographic characteristics of participants (*n* = 271).

**Variables**		**N (%)**	**FSS score** **(M ± SD)**	***t*** **/F**	***P***
**Age (years)**					
	18–39	37 (13.7)	27.71 ± 9.38	−4.831	<0.001
	40–59	234 (86.3)	36.50 ± 11.46		
**Gender**					
	Male	143(52.8)	31.62 ± 11.04	−5.404	<0.001
	Female	128(47.2)	38.88 ± 11.04		
**Income Level (CNY/Month)**					
	≤3,000	202 (74.5)	36.58 ± 11.58	3.819	<0.001
	>3,000	69 (25.5)	30.55 ± 10.53		
**Education level**					
	Primary school	85 (31.4)	39.18 ± 11.56	7.593	<0.001
	Junior high school	82 (30.0)	36.26 ± 11.20		
	High school	56 (20.8)	31.84 ± 10.87		
	Diploma	24 (8.9)	30.38 ± 10.76		
	Bachelor and above	24 (8.9)	28.42 ± 9.50		
**Marital status**					
	Unmarried	14 (5.2)	39.43 ± 10.05	10.286	<0.001
	Married/Cohabiting	221 (81.5)	33.34 ± 10.75		
	Divorced	16 (5.9)	41.81 ± 13.04		
	Widowed	20 (7.4)	45.35 ± 13.20		
**Occupational status**					
	Unemployed	37 (13.7)	42.27 ± 10.95	9.821	<0.001
	Employed	204 (75.3)	33.51 ± 10.94		
	Retired	30 (11.0)	36.57 ± 13.44		
**Living situations**					
	Couple living together	112 (41.3)	35.20 ± 10.58	5.897	<0.001
	Living with children	126 (46.5)	33.03 ± 11.74		
	Living alone	21 (7.7)	43.14 ± 11.94		
	Others	12 (4.5)	40.58 ± 12.03		

### Severity of fatigue

The mean Fatigue Severity Scale (FSS) score was 35.04 ± 11.60, reflecting varying degrees of fatigue among participants. Of the total sample, 124 participants (45.8%) reported significant fatigue, with scores of ≥ 36 (Table 2).

**Table 2:** PSF in stroke survivors.

**Variables**	**Frequency (n)**	**Percentage (%)**
Post-stroke fatigue	124	45.8
Non-post-stroke fatigue	147	54.2

### Depression, QoL, and ADLs

The mean depression score was 7.63 ± 6.13. Specifically, 41.7% of participants reported no depression, while 25.1% experienced mild depression, 15.5% had moderate depression, 13.3% showed moderately severe depression, and 4.4% reported severe depression. For QoL, the mean score was 34.28 ± 9.51. Among participants, 24.4% reported low QoL, 57.2% reported medium QoL, and 18.4% reported high QoL. The mean score for activities of daily living (ADLs) was 77.79 ± 25.90. Of the total sample, 43.5% displayed intact ability, 27.3% had mild impairment, 17.3% had moderate impairment, 6.3% had severe impairment, and 5.6% exhibited extreme impairment in daily activities (Table 3).

**Table 3:** Depression level, QoL and ADLs among stroke survivors (*n* = 271).

**Variables**		**Number**	**Percentage (%)**
**Depression Level**			
	No Depression	113	41.7
	Mild depression	68	25.1
	Moderate depression	42	15.5
	Moderately severe depression	36	13.3
	Severe depression	12	4.4
**Quality of life** ** (QoL)**			
	Low quality of life	66	24.4
	Medium quality of life	155	57.2
	High quality of life	50	18.4
**Activities of daily living (ADLs)**			
	Intact ability	118	43.5
	Mildly impaired	74	27.3
	Moderately impaired	47	17.3
	Severely impaired	17	6.3
	Extremely impaired	15	5.6

### Factors associated with fatigue severity

Multiple linear regression analysis revealed that age, gender, income level, depression level, QoL, and ADLs were significantly associated with the severity of fatigue (*P* < 0.05). Among these variables, depression level had the strongest positive association with the severity of fatigue (*β* = 0.415), followed by age (*β* = 0.116) and gender. In contrast, income level, QoL, and ADLs were negatively associated with the severity of fatigue (Table 4).

**Table 4:** Multiple linear regression analysis of factors associated with PSF in stroke survivors (*n* = 271).

**Independent variables**		**Unstandardized coefficient** **B (S.E.)**	**Standardized coefficient** *β*	***t***	***P***
**Model**					
	Constant	41.668 (5.849)		7.124	<0 .001
	Age	0.188 (0.073)	0.116	2.590	0.010
**Gender**					
	Male	0			
	Female	3.290 (0.966)	0.142	3.407	<0.001
**Income Level (CNY/Month)**					
	≤3,000	0			
	>3,000	−3.278 (1.349)	−0.123	−2.430	0.016
**Education level**					
	Primary school	0			
	Junior high school	−0.563 (1.189)	−0.022	−0.473	0.636
	High school	−0.668 (1.517)	−0.023	−0.440	0.660
	Diploma	−1.412 (2.198)	−0.035	−0.643	0.521
	Bachelor and above	−0.777 (1.897)	−0.019	−0.410	0.682
**Marital status**					
	Unmarried	0			
	Married / Cohabiting	−3.742 (3.240)	−0.125	−1.155	0.249
	Divorced	0.971 (2.797)	0.020	0.347	0.729
	Widowed	1.739 (2.966)	0.039	0.586	0.558
**Occupational status**					
	Unemployed	0			
	Employed	−2.332 (1.400)	−0.087	−1.666	0.097
	Retired	−1.740 (2.038)	−0.047	−0.854	0.394
**Living situations**					
	Couple living together	0			
	Living with children	−1.153 (1.125)	−0.050	−1.025	0.306
	Living alone	−0.854 (2.808)	−0.020	−0.304	0.761
	Others	0.826 (3.251)	0.015	0.254	0.800
**PHQ-9 scores**		0.786 (0.078)	0.415	10.113	<0.001
**SV-SS-QoL scores**		−0.255 (0.050)	−0.209	−5.121	<0.001
**BI scores**		−0.101 (0.019)	−0.226	−5.418	<0.001
		**R** ^**2**^	0.654		
		**Adjusted R** ^**2**^	0.629		
		**F**	26.414		

### Correlations between severity of fatigue and other factors

Pearson correlation analysis showed significant relationships between the severity of fatigue and demographic factors, as well as health-related variables. The severity of fatigue was positively correlated with age (*r* = 0.300, *P* < 0.01), gender (*r* = 0.313, *P* < 0.01), and marital status (*r* = 0.237, *P* < 0.01). Negative correlations were observed with income level (*r* =  − 0.227, *P* < 0.01), educational level (*r* =  − 0.314, *P* < 0.01), and occupational status (*r* =  − 0.142, *P* < 0.05). Additionally, the severity of fatigue showed a strong positive correlation with depression level (*r* = 0.616, *P* < 0.01) and significant negative correlations with QoL (*r* =  − 0.432, *P* < 0.01) and ADLs (*r* =  − 0.512, *P* < 0.01) (Table 5). These findings highlight the complex interplay of demographic and clinical factors associated with the severity of fatigue in stroke survivors.

**Table 5:** The correlations among variables (*n* = 271).

	**Age**	**Gender**	**Income**	**Education level**	**Marital status**	**Occupational status**	**Living situations**	**FSS scores**	**PHQ-9 scores**	**SV-SS-QoL scores**	**BI scores**
**Age**	1										
**Gender**	0.051	1									
**Income Level (CNY/Month)**	−0.116	0.177^**^	1								
**Education level**	−0.299^**^	0.036	0.514^**^	1							
**Marital status**	0.219^**^	0.229^**^	0.004	−0.087	1						
**Occupational status**	0.009	0.109	0.320^**^	0.276^**^	0.145^*^	1					
**Living situations**	−0.054	0.063	0.099	−0.066	0.210^**^	−0.112	1				
**FSS scores**	0.300^**^	0.313^**^	−0.227^**^	−0.314^**^	0.237^**^	−0.142^*^	0.117	1			
**PHQ-9 scores**	0.058	0.230^**^	−0.106	−0.112	0.090	−0.057	0.003	0.616^**^	1		
**SV-SS-QoL scores**	−0.223^**^	−0.193^**^	−0.024	0.108	−0.070	0.049	0.055	−0.432^**^	−0.223^**^	1	
**BI scores**	−0.151^*^	−0.202^**^	0.048	0.196^**^	−0.126^*^	0.118	−0.140^*^	−0.512^**^	−0.331^**^	0.251^**^	1

**Notes.**

* *P* < 0.05.

** *P* < 0.01.

FSS Fatigue Severity Scale PHQ-9 Patient Health Questionnaire-9 SV-SS-QoL Short Version of the Stroke Specific Quality of Life Scale BI Barthel index

## Discussion

This study systematically analyzed the multiple factors associated with PSF, including age, gender, income, depression level, QoL, and ADLs, while further exploring in depth the associations between depression, QoL, ADL, and PSF, along with their potential mechanisms.

The findings of this study indicate a significant association between age and fatigue severity, with middle-aged stroke survivors exhibiting more pronounced fatigue symptoms than their younger counterparts. This disparity is primarily attributed to age-related physiological decline, including reduced cardiovascular and pulmonary function, which limits recovery capacity and increases energy expenditure during daily activities (Saxon et al., 2021; Huang et al., 2024). Moreover, middle-aged survivors often face greater psychological burdens related to family and financial responsibilities, potentially triggering chronic inflammation and exacerbating fatigue (Jang et al., 2021; Liu et al., 2024). In contrast, younger stroke survivors, while less impacted by physiological deterioration, are susceptible to PSF due to sedentary lifestyles, high occupational stress, and irregular sleep patterns (Yan, Rau & Zhong, 2020). These findings underscore the critical need for age-specific interventions to address the distinct challenges faced by stroke survivors of different age groups. For middle-aged survivors, strategies such as cardiovascular monitoring, stress management programs, and comprehensive rehabilitation approaches may be particularly effective in mitigating fatigue and improving recovery outcomes. In contrast, younger survivors might benefit more from tailored lifestyle education aimed at promoting physical activity, maintaining regular sleep patterns, and achieving balanced nutrition.

Gender differences are significantly associated with the severity of fatigue, with female stroke survivors experiencing more severe fatigue compared to their male counterparts. This disparity may be linked to hormonal changes during menopause, which elevate inflammatory cytokine levels and disrupt sleep (Thomas et al., 2022). Moreover, societal expectations and caregiving responsibilities often expose women to heightened psychological stress, further intensifying PSF symptoms (Comer et al., 2024; Tziaka et al., 2024). In contrast, male survivors may underreport their symptoms due to cultural norms that discourage the expression of emotional or physical distress, leading to delays in diagnosis and intervention (Kutlubaev et al., 2013; Nadarajah & Goh, 2015). To address these disparities, gender-sensitive interventions are essential: women should receive psychological counseling, hormonal management, and stress reduction techniques, while men may benefit from education programs aimed at improving symptom recognition and fostering open communication about emotional well-being.

Income level emerged as another critical factor associated with PSF. Lower-income stroke survivors face a higher risk of severe fatigue due to limited access to healthcare, rehabilitation services, and mental health support (Sauvé-Schenk et al., 2024). Financial instability can exacerbate psychological stress, reduce recovery motivation, and lead to nutritional deficiencies that impair energy metabolism (Singh et al., 2020). By contrast, higher-income survivors benefit from better access to quality medical care, psychological support, and nutritional interventions, which collectively alleviate fatigue symptoms and improve recovery outcomes (Kayola et al., 2023; Liu et al., 2024). These findings underscore the importance of policy-level interventions, such as financial subsidies for rehabilitation programs, the establishment of community-based mental health services, and improved access to affordable nutritional support, to alleviate the burden of PSF among low-income populations.

Additionally, this study reveals the complex and interdependent relationships between depression, QoL, ADLs, and PSF. A bidirectional interaction exists between PSF and depression: persistent fatigue exacerbates depressive symptoms, such as anxiety, loss of interest, and social withdrawal, while depression, in turn, diminishes survivors’ motivation, restricts their participation in rehabilitation activities, and increases psychological distress, further aggravating PSF (Matoso et al., 2020; Pedersen et al., 2022). Moreover, PSF significantly impairs QoL, particularly in emotional and social domains. Fatigue reduces life satisfaction, heightens psychological stress, and diminishes engagement in meaningful activities, thereby eroding overall well-being (Rodgers et al., 2021; Nindorera et al., 2022). Furthermore, PSF adversely affects survivors’ functional abilities, limiting their capacity to perform essential ADLs such as dressing, eating, and bathing. This loss of independence often intensifies feelings of helplessness and reliance on others, which further exacerbates depression and fatigue. The interplay among these factors creates a self-sustaining cycle that perpetuates physical, emotional, and functional decline, posing a significant barrier to recovery (Kirchberger et al., 2022; Li et al., 2023; Lohaus et al., 2023).

To address these challenges, a comprehensive intervention strategy is essential, focusing on psychological, functional, and social dimensions. Early psychological assessments, such as cognitive-behavioral therapy or counseling, combined with continuous mental health assessments for stroke survivors, can help alleviate depressive symptoms, reduce psychological distress, and disrupt the fatigue-depression cycle. Functional rehabilitation programs, including occupational and physical therapy, can restore ADLs, promote independence, and ease the burden on caregivers. Additionally, enhancing social support through peer programs, group therapy, and community engagement can strengthen emotional well-being, rebuild social connections, and improve QoL. By integrating these interventions, stroke survivors can experience significant relief from PSF and its associated challenges, facilitating their overall recovery and long-term well-being.

This study has several limitations. First, it only examined a limited set of factors associated with PSF and did not include critical clinical indicators, such as inflammatory biomarkers and stroke severity, which could provide a more comprehensive understanding of PSF’s mechanisms. Future research should integrate multidimensional clinical data to explore the underlying biological pathways of PSF. Second, the cross-sectional design limits causal inference and prevents the analysis of temporal dynamics in PSF progression. Longitudinal studies are recommended to track PSF onset, progression, and potential contributing factors over time. Lastly, the study sample was restricted to stroke survivors diagnosed within three months post-stroke, which may not capture the long-term trajectory of PSF. Future research should expand the sample to include chronic stroke survivors to enhance the generalizability and depth of the findings.

## Conclusions

This study found that PSF is common among stroke survivors, with 45.8% of participants reporting significant fatigue. The severity of fatigue was significantly associated with age, gender, income level, depression level, QoL, and ADLs. Specifically, fatigue showed a positive correlation with depression and negative correlations with QoL and ADL. Interventions for PSF should focus on managing depression, improving QoL, and restoring ADLs, with tailored, stratified support for middle-aged patients, women, and low-income groups. Future research should explore the biological mechanisms of PSF, conduct longitudinal studies, and develop multidimensional intervention strategies to effectively alleviate fatigue and improve the overall quality of life in stroke survivors.

##  Supplemental Information

10.7717/peerj.19052/supp-1 Supplemental Information 1 Associated factors of post-stroke fatigue in stroke survivors

10.7717/peerj.19052/supp-2 Supplemental Information 2 Questionnaires

10.7717/peerj.19052/supp-3 Supplemental Information 3 STROBE Checklist

## Additional Information and Declarations

### Data Availability

The following information was supplied regarding data availability: The raw measurements are available in the Supplementary File.
