# Supplementary material for: Factors associated with post-stroke fatigue among stroke survivors: a cross-sectional study
Source: PeerJ. 2025 Mar 6;13:e19052. doi: 10.7717/peerj.19052 (PMC11890295; doi:10.7717/peerj.19052)
Supplement: Supplemental Information 2 [file peerj-13-19052-s002.docx]

**QUESTIONNAIRES**

**SOCIODEMOGRAPHIC CHARACTERISTICS**

Please fill in the blank or tick "√" in the box provided:

**A1. Age______ (years)**

**A2.** **Gender**

🗆 Male

🗆 Female

**A3. Income (CNY/ month)**

🗆 < 1000

🗆 1000-3000

🗆 > 3000

**A4. Education level**

🗆 Primary school

🗆 Junior high school

🗆 High school

🗆 Diploma

🗆 Bachelor and above

**A5. Marital status**

🗆 Unmarried

🗆 Married/Cohabiting

🗆 Divorced

🗆 Widowed

**A6. Occupational status**

🗆 Unemployed

🗆 Employed

🗆 Retired

**A7. Living situations**

🗆 Couple living together

🗆 Living with children

🗆 Living alone

🗆 Others

**调查问卷**

**一般人口学特征**

请在所提供的方框中画“√”：

**A1. 年龄_**_____

**A2. 性别**

🗆 男

🗆 女

**A3. 收入(元/月)**

🗆 <1000

🗆 1000-3000

🗆 ＞3000

**A4.** **教育程度**

🗆 小学

🗆 初中

🗆 高中

🗆 大专

🗆 本科及以上

**A5.** **婚姻状况**

🗆 未婚

🗆 已婚/同居

🗆 离异

🗆 丧偶

**A6.** **职业状况**

🗆 失业

🗆 在职

🗆 退休

**A7.** **居住情况**

🗆 夫妻同住

🗆 与子女同住

🗆 独居

🗆 其他

**FATIGUE SEVERITY SCALE** (**FSS**)

Please put a "√" in the box that best reflects your level of fatigue.

Notes: 1= Completely disagree, 2 = Disagree, 3 = Slightly disagree, 4 = Neutral, 5 = Slightly agree, 6 = Agree, 7 = Fully agree

| **Questions** | **Agreement Score** | | | | | | |
| --- | --- | --- | --- | --- | --- | --- | --- |
| B1. My motivation is lower when I am fatigued. | 1 | 2 | 3 | 4 | 5 | 6 | 7 |
| B2. Exercise brings on my fatigue. | 1 | 2 | 3 | 4 | 5 | 6 | 7 |
| B3. I am easily fatigued. | 1 | 2 | 3 | 4 | 5 | 6 | 7 |
| B4. Fatigue interferes with my physical functioning. | 1 | 2 | 3 | 4 | 5 | 6 | 7 |
| B5. Fatigue causes frequent problems for me. | 1 | 2 | 3 | 4 | 5 | 6 | 7 |
| B6. My fatigue prevents sustained physical functioning. | 1 | 2 | 3 | 4 | 5 | 6 | 7 |
| B7. Fatigue interferes with carrying out certain duties and responsibilities. | 1 | 2 | 3 | 4 | 5 | 6 | 7 |
| B8. Fatigue is among my three most disabling symptoms. | 1 | 2 | 3 | 4 | 5 | 6 | 7 |
| B9. Fatigue interferes with my work, family, or social life. | 1 | 2 | 3 | 4 | 5 | 6 | 7 |
| **Total Score: ______** | | | | | | | |
|  |  |  |  |  |  |  |  |

**疲劳严重度量表**

请在最能反映您疲劳程度的方框里画“√”。

说明：1 = 完全不同意；2 = 不同意；3 = 有点儿不同意；4 = 中立；5 = 有点儿同意；6 = 同意；7 = 完全同意

| **问题** | **得分** | | | | | | |
| --- | --- | --- | --- | --- | --- | --- | --- |
| B1. 当我感到疲劳时，我什么事都不想做。 | 1 | 2 | 3 | 4 | 5 | 6 | 7 |
| B2. 运动会使我感到疲劳。 | 1 | 2 | 3 | 4 | 5 | 6 | 7 |
| B3. 我很容易疲劳。 | 1 | 2 | 3 | 4 | 5 | 6 | 7 |
| B4. 疲劳影响我的体能。 | 1 | 2 | 3 | 4 | 5 | 6 | 7 |
| B5. 疲劳带来频繁的不适。 | 1 | 2 | 3 | 4 | 5 | 6 | 7 |
| B6. 疲劳使我不能保持体能。 | 1 | 2 | 3 | 4 | 5 | 6 | 7 |
| B7. 疲劳影响我从事某些工作。 | 1 | 2 | 3 | 4 | 5 | 6 | 7 |
| B8. 疲劳是最影响我活动能力的症状之一。 | 1 | 2 | 3 | 4 | 5 | 6 | 7 |
| B9. 疲劳影响我的工作、家庭或社会生活。 | 1 | 2 | 3 | 4 | 5 | 6 | 7 |
| **总分：______** | | | | | | | |
|  |  |  |  |  |  |  |  |

**PATIENT HEALTH QUESTIONNAIRE-9 (PHQ-9)**

In the past 2 weeks, how often have you been troubled by the following questions? Please put a "√" in the box that best reflects your health.

Notes: 0 =Not at all, 1 = Several days, 2 = More than half the days, 3 = Nearly every day

| **Questions** | **Score** | | | |
| --- | --- | --- | --- | --- |
| C1. Little interest or pleasure in doing things | 0 | 1 | 2 | 3 |
| C2. Feeling down, depressed, or hopeless | 0 | 1 | 2 | 3 |
| C3. Trouble falling or staying asleep, or sleeping too much | 0 | 1 | 2 | 3 |
| C4. Feeling tired or having little energy | 0 | 1 | 2 | 3 |
| C5. Poor appetite or overeating | 0 | 1 | 2 | 3 |
| C6. Feeling bad about yourself or that you are a failure or have let yourself or your family down | 0 | 1 | 2 | 3 |
| C7. Trouble concentrating on things, such as reading the newspaper or watching television | 0 | 1 | 2 | 3 |
| C8. Moving or speaking so slowly that other people could have noticed? Or the opposite — being so fidgety or restless that you have been moving around a lot more than usual | 0 | 1 | 2 | 3 |
| C9. Thoughts that you would be better off dead or hurting yourself in some way | 0 | 1 | 2 | 3 |
| **Total Score: ______** | | | | |

患者健康问卷-9

在过去2周内，您被以下问题困扰的频率有多少？请在最能反映您健康状况的方框里画“√”。

说明：0 = 完全没有；1 = 几天；2 = 超过一半的天数；3 = 几乎每天

| **问题** | **没有** | **有几天** | **一半以上时间** | **几乎每天** |
| --- | --- | --- | --- | --- |
| C1. 对做事缺乏兴趣或乐趣 | 0 | 1 | 2 | 3 |
| C2. 感到情绪低落、沮丧或绝望 | 0 | 1 | 2 | 3 |
| C3. 入睡困难、睡不安稳或睡眠过多 | 0 | 1 | 2 | 3 |
| C4. 感到疲倦或精力不足 | 0 | 1 | 2 | 3 |
| C5. 食欲不振或暴饮暴食 | 0 | 1 | 2 | 3 |
| C6. 觉得自己很糟或认为自己是个失败者，或让自己或家人失望 | 0 | 1 | 2 | 3 |
| C7. 对事物专注有困难，如阅读报纸、报纸或电视时难以集中注意力 | 0 | 1 | 2 | 3 |
| C8. 行动或说话如此缓慢，以至于其他人会注意到？或者相反--非常焦躁或坐立不安、动来动去的情况更胜于平常 | 0 | 1 | 2 | 3 |
| C9. 有不如死掉或以某种方式伤害自己的想法 | 0 | 1 | 2 | 3 |
| **总分：______** | | | | |

**SHORT VERSION OF THE STROKE SPECIFIC QUALITY OF LIFE SCALE** (**SV-SS-QoL**)

For each item, estimate your own quality of life by ticking (√) in the box corresponding to the answer that best reflects the degree to which you agree or disagree with each.

Notes: 1 = Strongly agree, 2 = Moderately agree, 3 = Neither agree nor disagree, 4 = Moderately disagree, 5 = Strongly disagree

| **Items** | | **Score** | | | | |
| --- | --- | --- | --- | --- | --- | --- |
| D1. Self-care | Did you need help taking a bath or shower? | 1 | 2 | 3 | 4 | 5 |
| D2. Mobility | Did you have to stop and rest more than you would like when walking or using a wheelchair? | 1 | 2 | 3 | 4 | 5 |
| D3. Upper extremity | Did you have trouble buttoning buttons? | 1 | 2 | 3 | 4 | 5 |
| D4. Language | Did you have to repeat yourself so others could understand you? | 1 | 2 | 3 | 4 | 5 |
| D5. Vision | Did you have trouble seeing the television well enough to enjoy a show? | 1 | 2 | 3 | 4 | 5 |
| D6. Work | Did you have trouble doing daily work around the house? | 1 | 2 | 3 | 4 | 5 |
| D7. Thinking | I had trouble remembering things. | 1 | 2 | 3 | 4 | 5 |
| D8. Family roles | I felt I was a burden to my family. | 1 | 2 | 3 | 4 | 5 |
| D9. Social roles | My physical condition interfered with my social life. | 1 | 2 | 3 | 4 | 5 |
| D10. Personality | My personality has changed. | 1 | 2 | 3 | 4 | 5 |
| D11. Mood | I was discouraged about my future. | 1 | 2 | 3 | 4 | 5 |
| D12. Energy | I was too tired to do what I wanted to do. | 1 | 2 | 3 | 4 | 5 |
| **Total Score:** | | | | | | |

**简化版脑卒中患者专门生活质量量表**

请在最能反映您生活质量的方框里画“√”。

说明：1 = 强烈同意;2 = 适度同意;3 = 既不同意也不反对;4 = 适度不同意,

5 = 强烈不同意

| **项目** | | **评分** | | | | |
| --- | --- | --- | --- | --- | --- | --- |
| D1. 自理 | 您洗澡时需要帮助吗？ | 1 | 2 | 3 | 4 | 5 |
| D2. 移动性 | 在行走或使用轮椅时，您是否经常需要停下来休息？ | 1 | 2 | 3 | 4 | 5 |
| D3. 上肢 | 扣纽扣时有困难吗？ | 1 | 2 | 3 | 4 | 5 |
| D4. 语言 | 您是否需要重复自己的话，以便他人能够理解您的意思？ | 1 | 2 | 3 | 4 | 5 |
| D5. 视力 | 您在看电视时能无障碍的欣赏完一个节目吗？ | 1 | 2 | 3 | 4 | 5 |
| D6. 工作 | 您在家做日常家务劳动是否有困难？ | 1 | 2 | 3 | 4 | 5 |
| D7. 思维 | 我记不住事情。 | 1 | 2 | 3 | 4 | 5 |
| D8. 家庭角色 | 我觉得自己是家庭的负担。 | 1 | 2 | 3 | 4 | 5 |
| D9. 社会角色 | 我的身体状况影响了我的社交生活。 | 1 | 2 | 3 | 4 | 5 |
| D10. 性格 | 我的性格发生了变化。 | 1 | 2 | 3 | 4 | 5 |
| D11. 情绪 | 我对自己的未来感到灰心丧气。 | 1 | 2 | 3 | 4 | 5 |
| D12. 精力 | 我太累了，不能做我以前想做的事。 | 1 | 2 | 3 | 4 | 5 |
| **总分:** | | | | | | |

**THE BARTHEL INDEX (BI)**

This scale is used to assess your self-care ability. For each item, please fill in the score that best reflects your own situation in the corresponding space.

Notes: 100 = Intact ability, 61~ 99 = Mildly impaired, 41~60 = Moderately impaired,

21~40 = Severely impaired, 20 = Extremely severely impaired

| **Activity** | **Items** | **Score** |
| --- | --- | --- |
| E1. Bowels | 0 = incontinent (or needs to be given enemas); |  |
|  | 5 = occasional accident; |  |
|  | 10 = continent. |  |
| E2. Bladder | 0 = incontinent, or catheterized and unable to manage alone; |  |
|  | 5 = occasional accident; |  |
|  | 10 = continent. |  |
| E3. Grooming | 0 = needs to help with personal care; |  |
|  | 5 = independent face/hair/teeth/shaving (implements provided). |  |
| E4. Bathing | 0 = dependent; |  |
|  | 5 = independent (or in shower). |  |
| E5. Toilet use | 0 = dependent E5; |  |
|  | 5 = needs some help, but can do something alone; |  |
|  | 10 = independent (on and off, dressing, wiping). |  |
| E6. Feeding | 0 = unable; |  |
|  | 5 = needs help cutting, spreading butter, etc., or requires modified diet; |  |
|  | 10 = independent. |  |
| E7. Dressing | 0 = dependent; |  |
|  | 5 = needs help but can do about half unaided; |  |
|  | 10 = independent (including buttons, zips, laces, etc.). |  |
| E8. Transfers  (Bed to chair and back) | 0 = unable, no sitting balance; |  |
|  | 5 = major help (one or two people, physical), can sit; |  |
|  | 10 = minor help (verbal or physical); |  |
|  | 15 = independent. |  |
| E9. Mobility  (On level surfaces) | 0 = immobile or < 50 yards; |  |
|  | 5 = wheelchair independent, including corners, > 50 yards; |  |
|  | 10 = walks with help of one person (verbal or physical) > 50 yards; |  |
|  | 15 = independent (but may use any aid; for example, stick) > 50 yards. |  |
| E10. Stairs | 0 = unable; |  |
|  | 5 = needs help (verbal, physical, carrying aid); |  |
|  | 10 = independent. |  |
| **Total Score: ______** | | |

**Barthel 量表**

请在相应空格里填写最能反映您日常生活活动能力的分数。

说明：100 = 完整；61-99 = 轻度受损；41-60 = 中度受损; 21-40 = 严重受损;

20 = 极度严重受损

| **项目** | **内容** | **得分** |
| --- | --- | --- |
| E1. 大便 | 0 = 失禁； |  |
|  | 5 = 偶尔失禁或需要器具帮助； |  |
|  | 10 = 能控制；如果需要，能使用灌肠剂或栓剂。 |  |
| E2. 小便 | 0 = 失禁； |  |
|  | 5 = 偶尔失禁或需要器具帮助； |  |
|  | 10 = 能控制；如果需要，能使用集尿器。 |  |
| E3. 修饰 | 0 = 需要帮助； |  |
|  | 5 = 独立洗脸、洗头、刷牙、剃须。 |  |
| E4. 洗澡 | 0 = 依赖； |  |
|  | 5 = 自理。 |  |
| E5. 入厕 | 0 = 依赖别人； |  |
|  | 5 = 需要部分帮助；在穿脱衣裤或使用卫生纸时需要帮助； |  |
|  | 10 = 独立用厕所或便盆，穿脱衣裤，冲洗或清洗便盆。 |  |
| E6. 吃饭 | 0 = 依赖别人； |  |
|  | 5 = 需要部分帮助（如切割食物，搅拌食物）； |  |
|  | 10 = 能使用任何需要的装置，在适当的时间内独立进食。 |  |
| E7. 穿衣 | 0 = 依赖； |  |
|  | 5 = 需要帮助，但在适当的时间内至少完成一半的工作； |  |
|  | 10 = 自理（系、开纽扣，关、开拉锁和穿脱支具）。 |  |
| E8. 转移 | 0 = 完全依赖别人，不能坐 ； |  |
|  | 5 = 能坐，但需要大量帮助（2）人才能转移 |  |
|  | 10 = 需少量帮助（1）人或指导 |  |
|  | 15 = 独立从床到轮椅，再从轮椅到床，包括从床上坐起、刹住轮椅、抬起脚踏板 |  |
| E9. 行走 | 0 = 不能动 |  |
|  | 5 = 在轮椅上独立行动，能行走 45 米 |  |
|  | 10 = 需要 1 人帮助行走（体力或语言指导）45 米 |  |
|  | 15 = 能在水平路面上行走 45 米，可以使用辅助装置，不包括带的助行器 |  |
| E10. 上下楼梯 | 0 = 不能 |  |
|  | 5 = 需要帮助和监督 |  |
|  | 10 = 独立，可以使用辅助装置 |  |
| **总分： ­­­** | | |
